# Metformin‐mediated increase in DICER1 regulates microRNA expression and cellular senescence

**DOI:** 10.1111/acel.12469

**Published:** 2016-03-17

**Authors:** Nicole Noren Hooten, Alejandro Martin‐Montalvo, Douglas F. Dluzen, Yongqing Zhang, Michel Bernier, Alan B. Zonderman, Kevin G. Becker, Myriam Gorospe, Rafael de Cabo, Michele K. Evans

**Affiliations:** ^1^ Laboratory of Epidemiology and Population SciencesNational Institute on AgingNational Institutes of Health251 Bayview BoulevardBaltimoreMD21224USA; ^2^ Translational Gerontology BranchNational Institute on AgingNational Institutes of Health251 Bayview BoulevardBaltimoreMD21224USA; ^3^ Pancreatic Islet Development and Regeneration UnitDepartment of Stem CellsCABIMER‐Andalusian Center for Molecular Biology and Regenerative MedicineAvenida Americo Vespucio, Parque Científico y Tecnologico Cartuja 9341092SevillaSpain; ^4^ Laboratory of GeneticsNational Institute on AgingNational Institutes of Health251 Bayview BoulevardBaltimoreMD21224USA

**Keywords:** aging, AUF1, caloric restriction, diabetes mellitus, microRNA, RNA‐binding proteins

## Abstract

Metformin, an oral hypoglycemic agent, has been used for decades to treat type 2 diabetes mellitus. Recent studies indicate that mice treated with metformin live longer and have fewer manifestations of age‐related chronic disease. However, the molecular mechanisms underlying this phenotype are unknown. Here, we show that metformin treatment increases the levels of the microRNA‐processing protein DICER1 in mice and in humans with diabetes mellitus. Our results indicate that metformin upregulates DICER1 through a post‐transcriptional mechanism involving the RNA‐binding protein AUF1. Treatment with metformin altered the subcellular localization of AUF1, disrupting its interaction with *DICER1* mRNA and rendering *DICER1* mRNA stable, allowing DICER1 to accumulate. Consistent with the role of DICER1 in the biogenesis of microRNAs, we found differential patterns of microRNA expression in mice treated with metformin or caloric restriction, two proven life‐extending interventions. Interestingly, several microRNAs previously associated with senescence and aging, including miR‐20a, miR‐34a, miR‐130a, miR‐106b, miR‐125, and let‐7c, were found elevated. In agreement with these findings, treatment with metformin decreased cellular senescence in several senescence models in a DICER1‐dependent manner. Metformin lowered p16 and p21 protein levels and the abundance of inflammatory cytokines and oncogenes that are hallmarks of the senescence‐associated secretory phenotype (SASP). These data lead us to hypothesize that changes in DICER1 levels may be important for organismal aging and to propose that interventions that upregulate DICER1 expression (e.g., metformin) may offer new pharmacotherapeutic approaches for age‐related disease.

## Introduction

Metformin has been used to treat type 2 diabetes mellitus since the 1950s. Metformin enhances insulin sensitivity and lowers blood glucose levels by inhibiting gluconeogenesis in the liver. Metformin elicits these effects by inhibiting the mitochondrial respiratory‐chain complex I and glycerophosphate dehydrogenase (mGPD) and also by activating adenosine monophosphate‐activated protein kinase (AMPK) (Foretz *et al*., 2014; Madiraju *et al*., 2014). Activation of AMPK, in turn, leads to a plethora of signaling cascades that regulate energy homeostasis and metabolism (Hardie *et al*., 2012). However, AMPK‐independent pathways activated by metformin have also been described (Pollak, 2012; Foretz *et al*., 2014) and recent data also suggest that systemic effects of metformin may have different mechanisms of action in different tissues (Duca *et al*., 2015). Gaps in our knowledge about the mechanisms that underlie its therapeutic effects persist.

Numerous epidemiologic studies show that in addition to its role in modulating metabolic pathways, metformin also influences factors associated with cancer incidence and cancer mortality among diabetics treated with the drug (Pollak, 2012). These retrospective epidemiologic studies have led to intensive focus on the potential use of metformin as an anticancer therapeutic agent. Although many studies have focused on uncovering how metformin elicits anticancer effects, the exact mechanism of tumor inhibition remains unknown (Foretz *et al*., 2014). Studies using cancer cells in culture and murine tumor xenografts have found that metformin inhibits tumorigenesis by upregulating DICER1 (Blandino *et al*., 2012), a key enzyme that processes microRNAs (miRNAs). Therefore, it is possible that upregulation of DICER1 may in part mediate metformin's antitumorigenic effects. In general with few exceptions, cancer cells have decreased global miRNA expression (Lu *et al*., 2005; Kumar *et al*., 2007), leading to the hypothesis that upregulation of DICER1 may lead to increased miRNA expression, which would then contribute to lowering tumorigenic activity. Consistent with this idea, DICER1 expression is decreased in several types of human cancers (Bahubeshi *et al*., 2011; Foulkes *et al*., 2014).

Conditional loss of mouse *Dicer1* in skin or adipocytes induces DNA damage and activates a p19^Arf^‐p53 signaling pathway leading to increased cellular senescence (Mudhasani *et al*., 2008; Mori *et al*., 2012), indicating that modulation of DICER1 levels may influence aging‐related pathways. Additionally, *dicer* loss‐of‐function mutations in *Caenorhabditis elegans* reduce lifespan and homeostatic stress responses (Mori *et al*., 2012). We reported that miRNA levels decrease with advancing age in human peripheral blood mononuclear cells (PBMCs) and primate skeletal muscle, while others have found decreased levels of miRNAs with age in mouse brain and in *C. elegans* (Noren Hooten *et al*., 2010, 2013; Smith‐Vikos & Slack, 2012; Mercken *et al*., 2013). Given the conservation of miRNAs across species, and that a single miRNA can regulate *C. elegans* lifespan and predict the mortality of individual *C. elegans* (Smith‐Vikos & Slack, 2012), it is likely that miRNAs play an important role in regulating lifespan in humans and other species.

The fact that modulating DICER1 and miRNAs may be important for regulating lower organismal aging and our recent findings that metformin significantly increases the longevity of mice (Martin‐Montalvo *et al*., 2013) led us to examine mechanisms by which DICER1 expression is altered by metformin and the possible role of DICER1 in aging. As gene expression is affected post‐transcriptionally by noncoding RNAs and RNA‐binding proteins (RBPs), we hypothesized that post‐transcriptional mechanisms may play a role in modulating levels of DICER1 in response to metformin. Previous evidence indicated that the RBP AU‐rich element‐binding factor 1 (AUF1), also known as hnRNPD (heterogeneous nuclear ribonucleoprotein D), lowers the stability of *DICER1* mRNA through binding to multiple regions in the coding region and 3′‐untranslated region (UTR) of *DICER1* mRNA (Abdelmohsen *et al*., 2012). AUF1 comprises 4 isoforms (p37, p40, p42, and p45) all of which contain two RNA‐recognition motifs (RRMs) (White *et al*., 2013; Yoon *et al*., 2014). In general, cytoplasmic AUF1 promotes the decay of target mRNAs (White *et al*., 2013). However, new data using the PAR‐CLIP (photoactivatable ribonucleoside‐enhanced cross‐linking and immunoprecipitation) method indicate that AUF1 also affects mRNA translation and enhances the stability of a subset of transcripts (Yoon *et al*., 2014). Through these activities, AUF1 regulates cellular processes such as senescence, proliferation, and stress and immune responses that impact upon aging and age‐related diseases.

Here, we explored the role of DICER1 in mice chronically treated with metformin or dietary caloric restriction, two interventions that extend mouse lifespan. We found higher levels of DICER1 in mice treated with metformin or caloric restriction as well as in humans with diabetes mellitus on metformin. In contrast, *DICER1* mRNA levels decreased with age in untreated human subjects. We present evidence that metformin treatment prevents the interaction of the decay‐promoting RBP AUF1 with *DICER1* mRNA, resulting in increased expression of DICER1. Given that metformin inhibits cellular senescence only in the presence of DICER1, our results highlight a possible mechanism that may explain in part the anti‐aging effects of metformin and identify relevant pathways and targets for further investigation.

## Results

### Age and metformin treatment alter DICER1 levels

**Figure 1:**
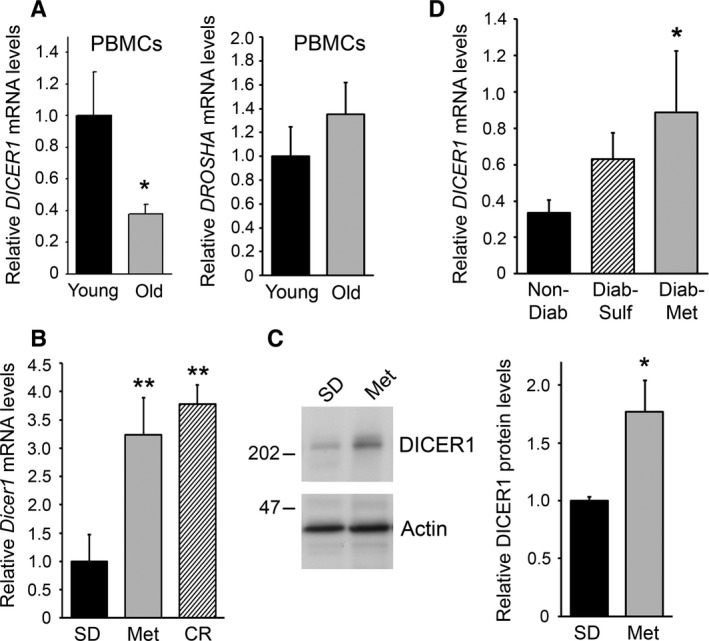
DICER1 levels are altered by human age and by metformin treatment in mice and humans. (A) *DICER1* and *DROSHA* mRNA levels were quantified from PBMCs from young (~30 years) and old (~64 years) individuals (*n* = 14/group) from the HANDLS study using RT–qPCR. (B) *DICER1* mRNA and DICER protein (C) levels were quantified by RT–qPCR and immunoblotting, respectively, from livers of mice on standard diet (SD), metformin‐treated (Met), or on calorie restriction (CR). For protein levels in C, a representative immunoblot is shown and Dicer levels from 8 mice per group were quantified from immunoblots and normalized to actin. (D) *DICER1* mRNA levels were quantified from PBMCs of nondiabetics, diabetics taking sulfonylureas, and diabetics taking metformin (*n* = 20/group). Demographic information for cohorts in (A) and (D) is in Table 1. The histograms represent the mean + SEM. *P < 0.05, **P ≤ 0.01.

We hypothesized that changes in DICER1 levels may contribute to the age‐associated decline in miRNAs (Noren Hooten *et al*., 2010). Therefore, we examined *DICER1* mRNA levels by reverse transcription (RT) followed by real‐time quantitative (q)PCR analysis from PBMCs from young (~30 year old) and older (~64 year old) individuals and found a significant decrease in *DICER1* mRNA levels with human age (Fig. 1A; Table 1A). In contrast, the levels of *DROSHA* mRNA, encoding another key protein in miRNA biosynthesis, were not significantly different with human age (Fig. 1A). Therefore, we focused on investigating whether changes in DICER1 levels may explain alterations in miRNA expression with age.

**Table 1:** Demographic information for human cohorts used in Fig. 1

A. Age cohort	Young	Old
*N*	14	14
Age (mean (SD))	30.1 (0.3)	64.2 (0.4)
Sex = Men (%)	6 (42.8)	6 (42.8)
Race = AA (%)	6 (42.8)	6 (42.8)
PovStat = Below (%)	6 (42.8)	5 (35.7)
BMIcut = Obese (%)	6 (42.8)	4 (28.6)

B. Diabetics cohort	Nondiabetics	Diabetics taking Sulfonylureas	Diabetics taking Metformin
*n*	20	20	20
Age (mean (SD))	49.45 (10.30)	52.15 (8.65)	50.50 (8.13)
Sex = Men (%)	7 (35.0)	7 (35.0)	7 (35.0)
Race = AA (%)	13 (65.0)	13 (65.0)	13 (65.0)
PovStat = Below (%)	7 (35.0)	9 (45.0)	6 (30.0)
BMIcut = Obese (%)	19 (95.0)	19 (95.0)	19 (95.0)

AA, African American; BMIcut, below or above obesity cut‐off of ≥ 30 kg m^−2^; PovStat, Poverty status, above or below the 125% Poverty line as defined by the Department of Health and Human Services in 2003.

Previously, metformin was found to increase the healthspan and longevity of mice (Martin‐Montalvo *et al*., 2013). Given that metformin elevates DICER1 in cultured cancer cells (Blandino *et al*., 2012), we wanted to examine whether changes in DICER1 expression occur in mice chronically treated with metformin and to compare these findings with those of mice on dietary caloric restriction, an intervention that extends lifespan in mice. In response to metformin and dietary caloric restriction, there is an upregulation in *Dicer1* mRNA levels in the livers of treated mice compared with control mice on standard diet (Fig. 1B), and livers of metformin‐treated mice had higher DICER1 protein levels than control mice (Fig. 1C).

Given that *DICER1* mRNA levels decreased in human PBMCs from older individuals compared with younger individuals (Fig. 1A, first panel) and that DICER1 levels increased by metformin treatment in mice, we investigated whether individuals being treated for diabetes with metformin also had higher levels of DICER1. Individuals in the Healthy Aging in Neighborhoods of Diversity across the Lifespan (HANDLS) study (Evans *et al*., 2010) were chosen based on the following criteria. The participants were either euglycemic normal controls (i), diabetics treated with sulfonylurea (ii), or diabetics treated with metformin (iii) (Table 1B). The sulfonylurea group was added in the study because diabetic individuals treated with metformin have increased survival compared with nondiabetics and with diabetics on sulfonylurea (Bannister *et al*., 2014). All groups were matched based on age, sex, race and body mass index (BMI) (*n* = 20 per group; Table 1B). After isolation of RNA from participant PBMCs and RT–qPCR analysis, diabetics treated with metformin were found to have significantly higher levels of *DICER1* mRNA compared with nondiabetics; diabetics taking sulfonylureas also had higher levels of *DICER1* mRNA, but this difference was not significant (Fig. 1D).

### Metformin regulates the RBP AUF1 localization and binding to *DICER1* mRNA

**Figure 2:**
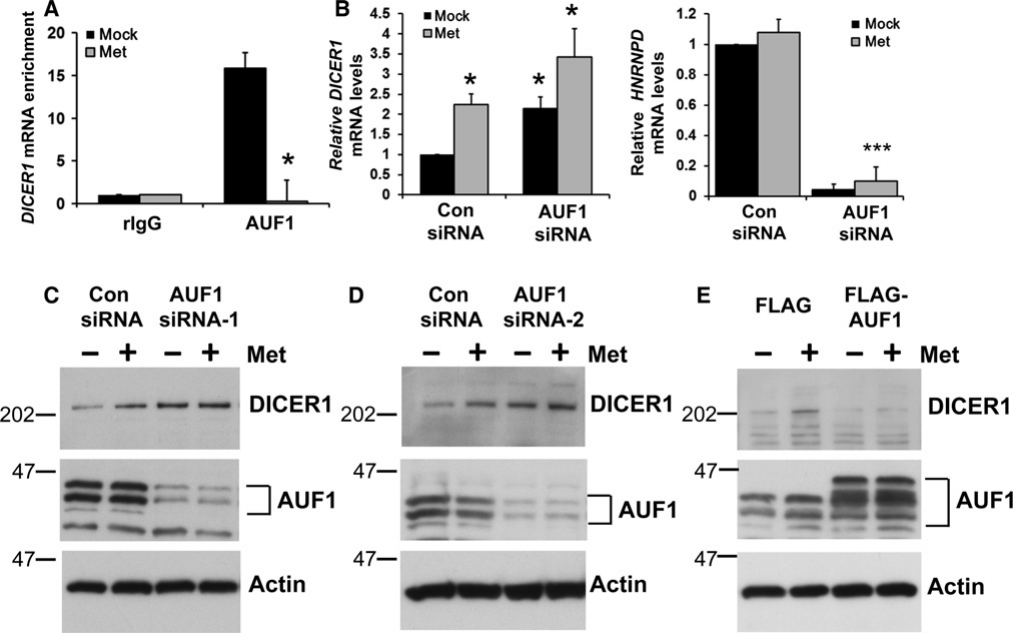
Metformin stabilizes *DICER1* expression through modulating the binding of the RNA‐binding protein AUF1. (A) Serum‐starved HepG2 cells were treated with 500 μm metformin or PBS for 1 h and ribonucleoprotein (RNP) immunoprecipitation (RIP) assays followed by RT–qPCR analysis was used to measure the enrichment of *DICER1* mRNA in AUF1 immunoprecipitates. Each IP was compared to control IgG IP and normalized to *GAPDH* mRNA levels. (B) HeLa cells were transfected with either AUF1 or Control (Con) siRNA and 24 h later, cells were treated with metformin for 24 h. The levels of *HNRNPD* mRNA (which encodes AUF1) and *DICER1* mRNA were measured by RT–qPCR analysis and normalized to *GAPDH* mRNA levels. (C–E) Cells were transfected with either AUF1 siRNA‐1 (Qiagen), AUF1 siRNA‐2 (Santa Cruz) or the indicated FLAG‐tagged plasmids were treated as in (B), and lysates were analyzed by immunoblotting to assess protein levels of DICER1, AUF1 and actin using specific antibodies. Histograms represent the mean + SEM from three independent experiments. ***P < 0.001 or *P < 0.05 by Student's *t*‐test.

Given that chronic metformin treatment in mice increased DICER1 levels, we hypothesized that metformin may alter post‐transcriptional processes that would affect the stability and/or turnover of *Dicer1* mRNA. In response to changes in the environment, RNA‐binding proteins (RBP) regulate gene expression by affecting the turnover and/or translation of bound mRNAs (Moore, 2005). Previous studies showed that the RBP AUF1 binds *DICER1* mRNA and negatively regulates DICER1 protein levels by lowering the stability of *DICER1* mRNA (Abdelmohsen *et al*., 2012). Therefore, we hypothesized that metformin may enhance DICER1 expression by altering the binding of AUF1 to *DICER1* mRNA. To test this possibility, we performed a ribonucleoprotein immunoprecipitation (RIP) assay to examine the effect of metformin on the AUF1‐*DICER1* mRNA complex. While AUF1 was found to bind *DICER1* mRNA under basal conditions, metformin treatment dramatically reduced this interaction (Fig. 2A). As AUF1 binding degrades *DICER1* mRNA, these data suggest that by triggering the dissociation of AUF1‐*DICER1* mRNA complexes, metformin treatment may stabilize *DICER1* mRNA. To further test this possibility, small interfering RNAs were used to downregulate AUF1 levels in cells. Lowering AUF1 increased *DICER1* mRNA and protein levels (Fig. 2B–D). *DICER1* mRNA levels further increased by metformin treatment, which may reflect an effect on *DICER1* at the transcriptional level (Fig. 2B). Conversely, AUF1 overexpression decreased DICER1 protein levels (Fig. 2E). These findings suggest that metformin treatment enhances DICER1 expression at least in part by dissociating AUF1 from *DICER1* mRNA.

**Figure 3:**
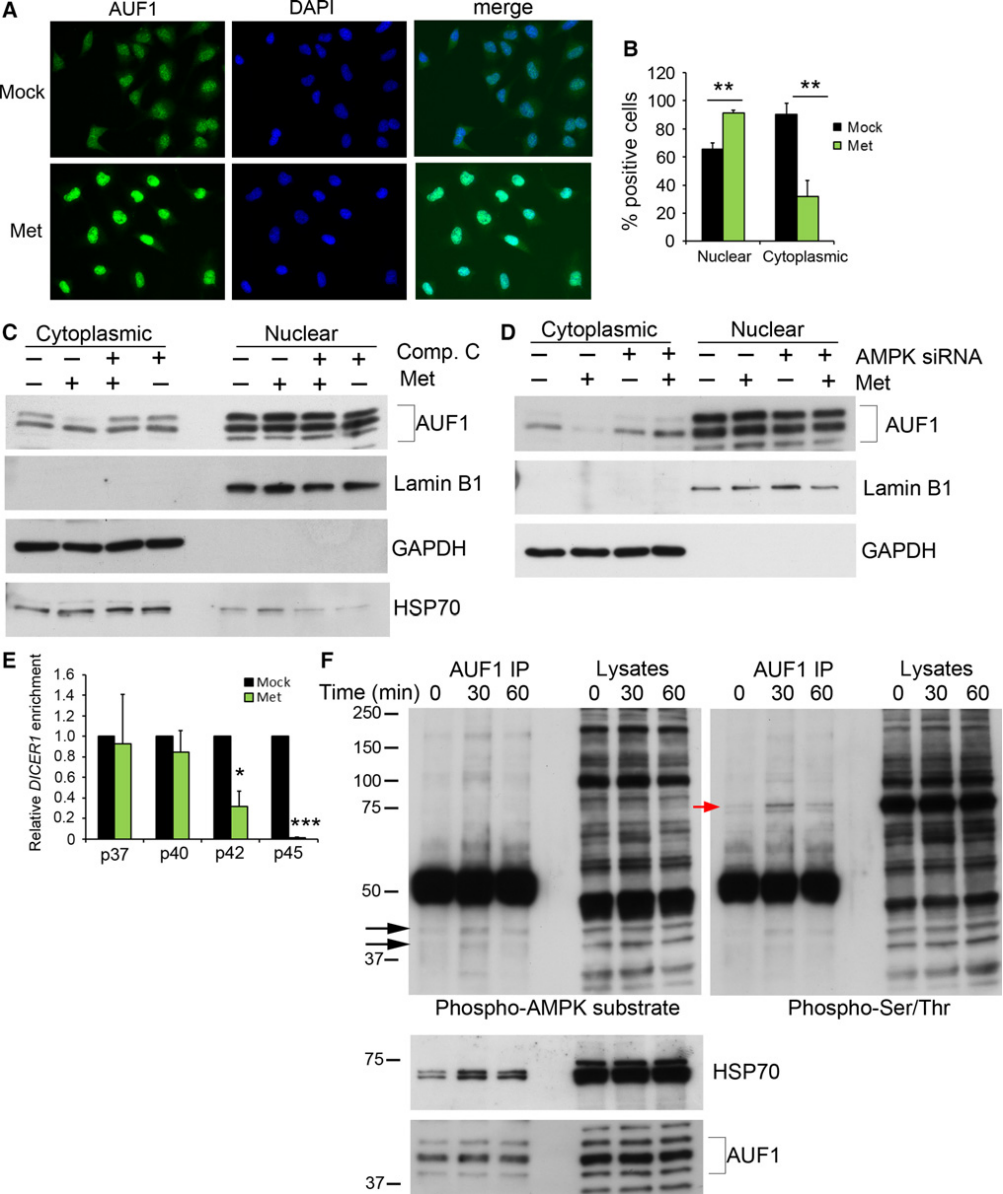
Metformin induces AUF1 nuclear retention. (A) Serum‐starved HeLa cells were treated with 500 μm metformin for 1 h. Cells were fixed and stained with an AUF1 antibody and DAPI. (B) AUF1 staining in the nucleus and cytoplasm was quantified and normalized to DAPI‐stained nuclei. (C) HeLa cells were pretreated with Compound C for 1 h or vehicle and were then subsequently treated with 500 μm metformin for 1 h. Cells were fractionated into cytoplasmic and nuclear fractions and analyzed by immunoblotting with anti‐AUF1, anti‐HSP70, anti‐Lamin B1 (nuclear marker), and anti‐GAPDH antibodies (cytoplasmic marker). (D) HeLa cells were transfected with AMPK siRNA or control and treated with metformin as described above. (E) HeLa cells transfected with individual isoforms of AUF1 were treated with or without metformin, and RIP assays were performed to measure the enrichment of *DICER1* mRNA in FLAG immunoprecipitates. Each IP was compared to control FLAG IP, normalized to *GAPDH* mRNA levels, and then normalized to mock‐treated cells. (F) AUF1 immunoprecipitates from HeLa cells treated with 500 μm metformin for the indicated time points were probed with anti‐phospho‐AMPK substrate, anti‐phospho‐serine/threonine, anti‐HSP70, and anti‐AUF1 antibodies. Black arrows indicate AUF1 phosphorylation, and red arrow indicates HSP70 phosphorylation. Histograms in B and E represent the mean + SEM from three independent experiments. *P < 0.05, **P < 0.01, ***P < 0.001 by Student's *t*‐test.

The abundance of AUF1 was not altered by metformin treatment of mice or cells in tissue culture (Fig. S1A,B); however, metformin affected AUF1 subcellular localization. Although AUF1 was found in both the cytoplasm and nucleus under basal conditions, by 1 h after addition of metformin, AUF1 had redistributed to a predominantly nuclear localization (Fig. 3A,B). This redistribution to the nucleus in response to metformin was confirmed by fractionating cells into the nuclear and cytoplasmic compartments in both HeLa and WI‐38 cells (Figs 3C, S1D). Given that AMPK is activated in response to metformin, we tested whether AMPK activation is important for the effects of metformin on AUF1 subcellular localization. Treatment with the AMPK inhibitor Compound C blocked AUF1 redistribution to the nucleus in response to metformin (Figs 3A, S1D), suggesting that the effects of metformin on AUF1 localization are influenced by AMPK. Furthermore, lowering AMPK levels using siRNA inhibited AUF1 redistribution from the cytoplasm to the nucleus in response to metformin (Fig. 3D). This translocation of AUF1 from the cytoplasm to the nucleus was associated with the release of AUF1‐*DICER1* mRNA complex, facilitating *DICER1* mRNA stabilization and translation.

The stoichiometry of the various AUF1 isoforms differed in their subcellular localization in response to metformin. Specifically, the localization of p45 was most affected by metformin, although p42/p40 isoforms (typically found together as a single band) were moderately affected as well. PAR‐CLIP data showed that the p40, p42, and p45 isoforms bind to *DICER1* mRNA; p45 in particular bound to multiple regions within *DICER1* mRNA (Table S1). Given this evidence, we tested whether metformin affected AUF1 isoform binding to *DICER1* mRNA. Each FLAG‐tagged AUF1 isoform was individually transfected into cells, and RIP assays were performed in control and metformin‐treated cells. As shown (Fig. 3E), metformin treatment did not affect p37 or p40 binding to *DICER1* mRNA, but it dramatically decreased *DICER1* mRNA bound to the p42 and p45 isoforms, supporting the notion that AUF1 translocation to the nucleus is associated with the dissociation of *DICER1* mRNA from AUF1.

We next investigated whether AUF1 is phosphorylated in response to metformin. Metformin treatment increased AUF1 phosphorylation as detected using antibodies that recognize the phosphorylated AMPK substrate motif and with phospho‐serine/threonine antibodies (Fig. 3F). Interestingly, several other proteins were prominent in AUF1 immunoprecipitates, among them a ~70‐kDa band. It was previously reported that AUF1 binds to HSP70 and that HSP70 shuttles AUF1 to the nucleus in response to heat shock (Laroia *et al*., 1999). Western blot analysis of HSP70 in the AUF1 immunoprecipitated material revealed that the AUF1‐HSP70 interaction was enhanced in the presence of metformin (Fig. 3F). Finally, HSP70 increased in the nucleus after metformin treatment (Fig. 3C). These data suggest that AUF1 translocation to the nucleus may be modulated by HSP70 binding in response to metformin.

### Metformin inhibits cellular senescence through DICER1

**Figure 4:**
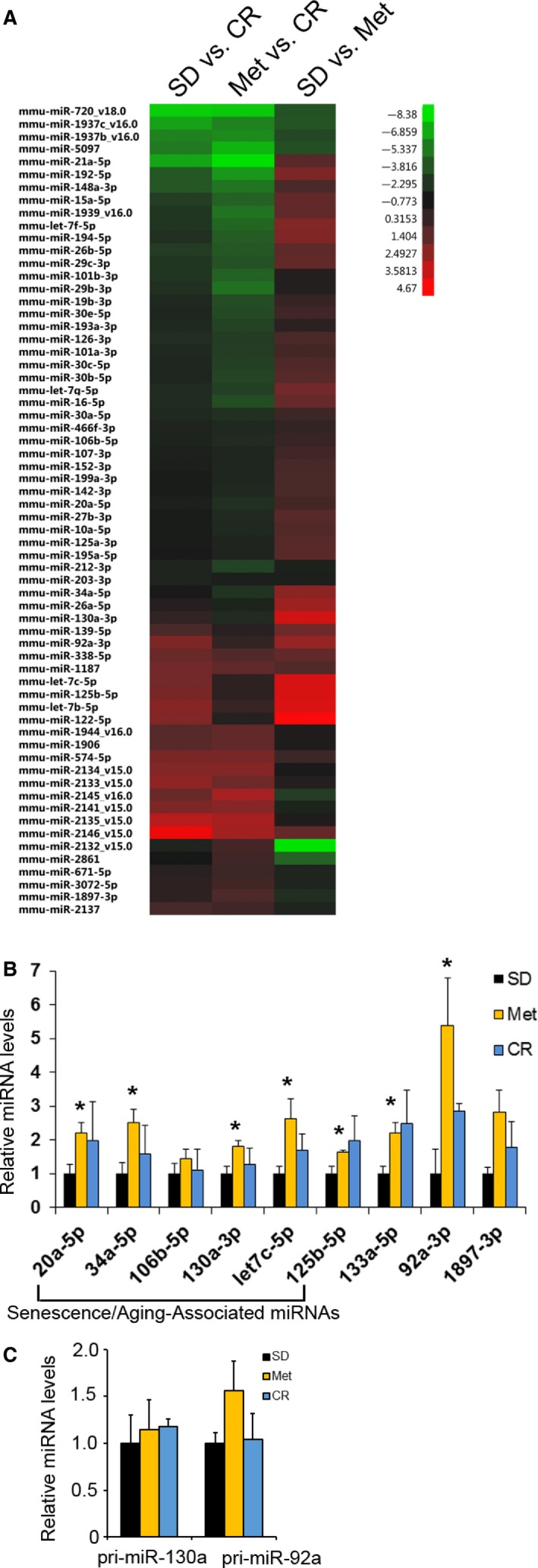
miRNA expression changes in mice treated with metformin or caloric restriction. (A) miRNAs were isolated from livers of mice on standard diet (SD), metformin (Met; 0.1%), or on caloric restriction (CR). Using total liver RNA, miRNA microarray analysis was performed. Heat map indicating miRNAs significantly changed in any of the indicated comparisons (z ratio). (B) RNA was isolated from livers from mice on standard diet (SD), treated with metformin (Met), or on caloric restriction (CR). Mature miRNA (B) or pri‐miRNA levels (C) were quantified by RT–qPCR analysis. The histograms represent the mean + SEM for SD (*n* = 5), Met (*n* = 5), and CR (*n* = 6). *P < 0.05 and **P < 0.01 compared with SD by Student's *t*‐test.

As the functional role of DICER1 in cells is to process miRNAs from precursors to mature miRNAs, it is likely that changes in DICER1 expression would ultimately alter miRNA expression. To test this idea, we analyzed global miRNA profiles using microarrays. Many miRNAs were significantly altered after metformin treatment or calorie restriction (Fig. 4A; Table S2). Consistent with the higher DICER1 levels in the liver of metformin‐treated mice, many miRNAs were found to be upregulated by metformin (Figs 4A,B, S2). To assess whether the increases in mature miRNAs were due to elevated transcription, we quantified the primary miRNA (pri‐miRNA, the initial unprocessed transcript of miRNAs) transcripts of two miRNAs observed to be differentially expressed in the presence of metformin, miR‐130a‐3p and miR‐92a‐3p (Fig. 4B,C). As shown, neither pri‐miR‐130a nor pri‐miR‐92a levels were significantly increased by metformin or CR (Fig. 4C). These data suggest that the increase in mature miRNAs in mice treated with metformin was not primarily a result of increased transcriptional activity.

**Figure 5:**
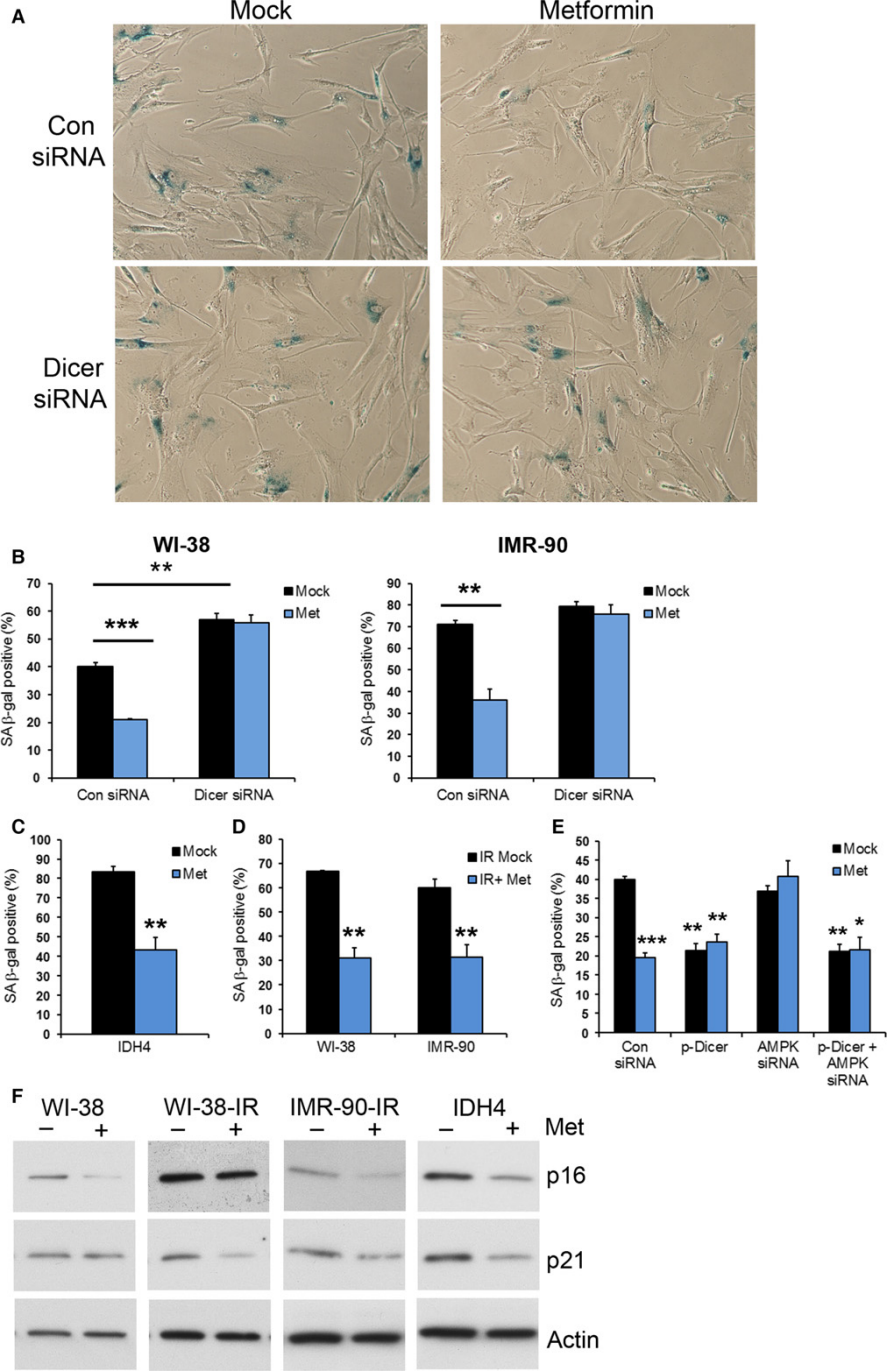
Inhibition of cellular senescence by metformin requires DICER1. Presenescent WI‐38 or IMR‐90 cells were transfected with either the indicated siRNAs or transfected with pDEST‐DICER1 and then either treated with PBS or with 500 μm metformin (Met) for 48 h and stained for SA‐β‐gal activity (A,B,D). (C) IDH4 cells cultured without dex were incubated with metformin. (D) WI‐38 and IMR‐90 cells exposed to ionizing radiation (IR) were treated with metformin. SA‐β‐gal‐positive cells were counted and normalized to the total number of cells. Representative phase‐contrast images from WI‐38 cells from (B) are shown, and the histograms represent the mean + SEM from 3 independent experiments. *P < 0.05, **P < 0.01 and **P < 0.01 by Student's *t*‐test. (F) Lysates from the indicated cell lines and treatments were analyzed for protein levels of senescence markers and actin for a protein loading control.

Several of the miRNAs upregulated by metformin (miR‐20a, miR‐30, miR‐34a, miR‐106b, miR‐130a/b, let‐7, miR‐125) are important for regulating cellular senescence or organismal aging (Fig. 4B). Furthermore, many of the targets of these miRNAs are important for regulating senescence (Table S3), and some have opposite expression patterns in response to metformin compared with senescence, suggesting that metformin may affect lifespan by inhibiting cellular senescence. To investigate this possibility, we examined whether metformin affects cellular senescence in culture using several different senescence models: WI‐38 human fetal lung diploid fibroblasts, IMR‐90 human skin fibroblasts, and IDH4 fibroblasts. We chose doses of metformin that were comparable to circulating drug levels of mice treated with 0.1% metformin (~0.45 mm; 74.3 mg L^−1^) and the therapeutic range in humans (1.64 ± 0.13 mg L^−1^ to 6.57 ± 0.61 mg L^−1^) (Sum *et al*., 1992). Metformin inhibited replicative senescence in WI‐38 and IMR‐90 cells (Fig. 5A,B,F). In IDH4 cells, senescence is induced by removal of dexamethasone (dex) (Wright *et al*., 1989). Dex was removed, and cells were cultured in the presence of metformin for 7 days; metformin treatment reduced the number of SA‐β‐gal‐positive IDH4 cells (Fig. 5C). Additionally, we induced senescence by exposing WI‐38 and IMR‐90 cells to ionizing radiation (IR). Treatment of cells with metformin delayed IR‐induced senescence, as assessed initially by SA‐β‐gal‐positive cells (Fig. 5D) and subsequently confirmed by examination of two well‐established protein markers of senescence, p16 and p21. All of these markers of senescence were decreased by metformin treatment (Fig. 5F).

**Figure 6:**
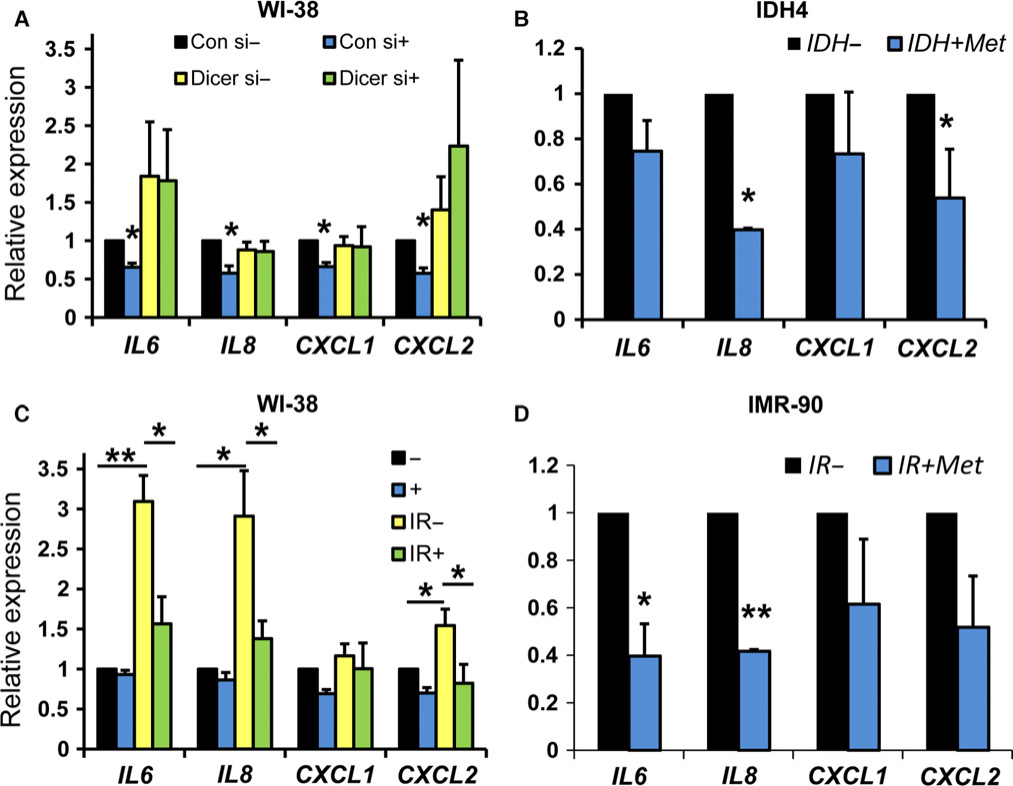
SASP mRNA levels are decreased by metformin. RNA was isolated from the indicated cell lines and treatments as detailed in Experimental procedures. mRNA levels of the indicated genes were quantified by RT–qPCR. IR, ionizing radiation; (−), mock‐treated (+), metformin‐treated.

A further hallmark trait of senescent cells is the secretion of inflammatory cytokines, chemokines, and oncogenes (the senescence‐associated secretory phenotype or SASP) (Coppe *et al*., 2008). We quantified the levels of several mRNAs encoding SASP factors in WI‐38 cells, in IDH4 cells grown without dex, and WI‐38 and IMR‐90 cells exposed to IR. The levels of *IL6, IL8, CXCL1* (which encodes GRO‐a), and *CXCL2* (which encodes GRO‐b) mRNAs decreased by metformin treatment (Fig. 6).

We next addressed whether the effects of metformin on senescence were dependent on DICER1. SiRNA‐mediated downregulation of DICER1 levels increased cellular senescence and blocked the ability of metformin to lower the number of SA‐β‐gal‐positive cells in both WI‐38 and IMR‐90 cells (Fig. 5A,B). Conversely, overexpression of DICER1 decreased cellular senescence (Fig. 5E). To determine whether AMPK is required for the senescence‐inducing effects of metformin, we silenced AMPK levels. This intervention blocked the metformin‐mediated decrease in SA‐β‐gal‐positive cells, and this effect was reversed with DICER1 overexpression. Collectively, these data indicate that metformin may reduce cellular senescence by promoting DICER1 expression and that AMPK is important for these effects.

## Discussion

Previously, we reported that chronic metformin treatment increases both healthspan and lifespan in mice (Martin‐Montalvo *et al*., 2013). Here, we have found that DICER1 levels are upregulated by metformin treatment in both mice and humans in part through a post‐transcriptional mechanism involving the RBP AUF1. Upregulation of DICER1 by metformin leads to higher levels of a subset of miRNAs important for regulating senescence and aging‐associated pathways. Furthermore, metformin treatment inhibits cellular senescence through a DICER1‐dependent mechanism.

Our data suggest that DICER1 levels may contribute to aging. Consistent results have been observed in a limited number of studies that have examined the role of DICER1 in aging. A study in primary rat cerebromicrovascular endothelial cells (CMVECs) found a decrease in DICER1 levels with age, with concomitant reduction in miRNA expression (Ungvari *et al*., 2013). Furthermore, aged mouse adipocytes also have lower DICER1 levels (Mori *et al*., 2012). Here, we found that *DICER1* mRNA levels decreased with age in human PBMCs, which may provide a mechanism for lower miRNA levels we previously observed in this cell population in the same cohort of individuals (Noren Hooten *et al*., 2010).

Compelling evidence suggests that alterations in DICER1 levels or activity may be important for other age‐associated diseases, especially cancer. In general, DICER1 levels are decreased in different types of human tumors and germline mutations in *DICER1* are associated with a rare type of childhood cancer called pleuropulmonary blastoma (PPB) (Bahubeshi *et al*., 2011; Foulkes *et al*., 2014). Point mutations in *DICER1* have also been described leading to tumor predisposition and referred to as *DICER1* syndrome (Bahubeshi *et al*., 2011; Foulkes *et al*., 2014). However, the role of DICER1 in cancer is complicated as mouse cancer models suggest that loss of one copy of the *Dicer1* gene increases tumorigenesis, whereas loss of both copies inhibits it, leading to the idea that DICER1 functions as a haploinsufficient tumor suppressor (Bahubeshi *et al*., 2011; Foulkes *et al*., 2014). These pieces of evidence may reflect the importance of DICER1 on cell survival and/or indicate that a subset of miRNAs is important for cancer progression. Nevertheless, these studies point to the importance of DICER1 and the need to fully understand the consequences of altering DICER1 levels and/or activity.

We found increased levels of miRNAs in livers of metformin‐treated mice, consistent with the fact that DICER1 processes precursor miRNAs to mature miRNAs. As DICER1 also plays other important roles in the RNA‐induced silencing complex (RISC) and in RNA metabolism (Burger & Gullerova, 2015), we cannot exclude the possibility that these other functions of DICER1 may be altered by metformin and contribute to metformin signaling. In addition, upregulation of DICER1 by metformin may also be regulated, in part, at the transcriptional level (Blandino *et al*., 2012); a comprehensive view of the spectrum of DICER1 functions influenced by metformin is beyond the scope of this investigation.

By examining the miRNAs that were upregulated by metformin treatment, we observed that several of these miRNAs have been implicated in regulating cellular senescence or aging (Table S3). Notably, we found that metformin treatment upregulated miR‐125, a putative homolog of the *C. elegans* miRNA lin‐4, which is a well‐established regulator of lifespan in worms (Boehm & Slack, 2005; Ibáñez‐Ventoso *et al*., 2008). Mutation of *dicer* decreases *C. elegans* longevity in response to heat stress, an effect that can be rescued by overexpression of lin‐4 (Mori *et al*., 2012). Here, the increase in miR‐125‐5p levels coincided with lifespan extension in mice in response to metformin (Boehm & Slack, 2005; Martin‐Montalvo *et al*., 2013). It will be interesting in future studies to examine whether miR‐125 has life‐extending properties, similar to those conferred to *C. elegans* by its homolog lin‐4.

Other miRNAs upregulated by metformin have also been linked to aging in model systems, including miR‐34. An age‐associated alteration in miR‐34 occurs in the mouse and overexpression of miR‐34 in *Drosophila* extends lifespan (Li *et al*., 2011; Liu *et al*., 2012). miR‐34 may play an important role in responding to DNA damage and also in protecting against osteoporosis and neurodegeneration (Kato *et al*., 2009; Liu *et al*., 2012; Krzeszinski *et al*., 2014). Metformin has been shown to upregulate miR‐34a in cancer cells in culture (Do *et al*., 2014). In addition to miR‐125 and miR‐34a, compelling evidence also suggests that other miRNAs including miR‐20a, miR‐30, miR‐106b, miR‐130a/b, and let‐7 family members are important regulators of cellular senescence (Table S3) (Abdelmohsen & Gorospe, 2015). With advancing age, the number of senescent cells increases and contributes to age‐related physiology and pathologies (van Deursen, 2014). We propose that one potential mechanism by which metformin increases lifespan is through inhibition of cellular senescence. Metformin decreases senescence in several different cell lines and senescence model systems. We provide evidence that metformin lowers protein levels of p16 and/or p21 and also decreases levels of *IL6, IL8, CXCL1* and *CXCL2* mRNAs, all encoding critical SASP factors. A previous report found that metformin enhances cellular senescence using different cell model systems and high doses of metformin (1–5 mm) with possible value in cancer therapy (Cufi *et al*., 2012). Here, we chose a lower range of concentrations (100–500 μm) that reflects the physiological circulating levels of metformin in mice and in diabetic patients treated with metformin (Sum *et al*., 1992).

We describe a post‐transcriptional mechanism whereby metformin treatment causes the redistribution of the RBP AUF1 from the cytoplasm to the nucleus, associated with a disruption of its interaction with *DICER1* mRNA that causes increased *DICER1* mRNA and protein levels. Cellular glucose levels also regulate AUF1 shuttling to the nucleus and AMPK is important for this effect (Gao *et al*., 2014). Here, we found that AMPK is also important in mediating the effects of metformin on AUF1 redistribution to the nucleus. Furthermore, metformin increased the phosphorylation of AUF1 and enhanced AUF1 binding to HSP70, an interaction that shuttles AUF1 to the nucleus in response to heat‐shock (Laroia *et al*., 1999). We found evidence that metformin enhanced AUF1 binding to HSP70 and increased HSP70 localization in the nucleus. Interestingly, a ~70 kDa band that was serine–threonine‐phosphorylated in AUF1 immunoprecipitates might represent HSP70, recently identified as a substrate of AMPK (Schaffer *et al*., 2015). It has been observed that phosphorylation of AUF1 has been reported to regulate AUF1 protein stability and binding to target mRNAs (Wilson *et al*., 2003; Li *et al*., 2013). AUF1 has also been implicated in regulating cellular senescence and also targets p16^INK4a^, a potent regulator of senescence (Guo *et al*., 2010; Pont *et al*., 2012). It will be interesting to determine whether other AUF1 targets are also affected by metformin.

Although the actions of metformin have been extensively studied for decades, the underlying mechanisms remain unclear. Here, we have extended our mouse lifespan studies by showing that chronic metformin treatment affects DICER1 levels both in mice and humans. This new insight will hopefully aid both in identifying novel targets for development of agents that could be important in the primary or secondary prevention of cancer and increasing healthspan in older individuals. Currently, the FDA is considering a clinical trial called Targeting Aging with Metformin (TAME), which aims to assess whether metformin can delay aging and age‐related diseases. Our studies provide timely insight into the molecular mechanisms through which metformin elicits anti‐aging effects in mice and humans.

## Experimental procedures

### Human study participants and mouse models

For Fig. 1A, a subcohort of young (30 years) and older (64 years) participants from the Healthy Aging in Neighborhoods of Diversity across the Life Span study (HANDLS) (Evans *et al*., 2010) were chosen for examination of *DICER1* and *DROSHA* mRNA levels with age. Clinical information on this subcohort has been described previously and is also listed in Table 1A (*n* = 14/group) (Noren Hooten *et al*., 2010) and explained in the Supporting Information. *DICER1* levels were quantified from PBMCs by RT–qPCR analysis as described below and in Supporting Information.

Frozen tissue from Martin‐Montalvo *et al*. was used for the current study (Animal protocol #: 352‐TGB‐2015). Livers from C57BL/6 mice on metformin (0.1% w/w in diet), calorie restriction (60% daily food, AIN‐93G, allotment compared with *ad lib* animals) or standard diet (AIN‐93G diet) used for these studies were described previously (Martin‐Montalvo *et al*., 2013).

Global microRNA expression from livers of the same cohort of mice (*n* = 5 per group) whose global gene expression profile was previously reported (Accesssion Number: GSE40936) (Martin‐Montalvo *et al*., 2013). Total RNA including miRNAs was isolated using the Absolutely RNA miRNA Kit (Agilent) and analyzed using the Agilent Mouse miRNA Microarray 15.0. Microarray was performed and analyzed as previously described (Noren Hooten *et al*., 2013). Individual miRNAs with pairwise z‐test *P* value ≤ 0.05, absolute value of *Z* ratio ≥ 1.5, with fdr ≤ 0.3 were considered significantly changed. The microRNA microarray data can be accessed at GEO (Accession Number: GSE73393). miRNA expression information from heat map is in Table S2.

### Cell lines, reagents, and transfections

HepG2 cells were maintained in Minimal Essential Media containing 10% fetal bovine serum (FBS) and HeLa, IMR‐90 and IDH4 cells were grown in Dulbecco's modified Eagle's medium (DMEM) supplemented with 10% FBS. IDH4 cells were further supplemented with 1 μg mL^−1^ dexamethasone (Wright *et al*., 1989). WI‐38 human fetal lung diploid fibroblasts (HDFs, Coriell Cell Repositories) were grown in DMEM supplemented with 10% FBS and 1% nonessential amino acids. All media contained penicillin/streptomycin. Metformin (Farmhispania S.A., Barcelona, Spain) was made fresh as a 100 mm stock solution in PBS. PBS was used as a vehicle control in all experiments. Compound C (Dorsomorphin) was purchased from Sigma‐Aldrich, St. Louis, MO, USA.

Cells were transfected using Lipofectamine 2000 (Invitrogen, Carlsbad, CA, USA) with siRNAs directed to *AUF1* (Qiagen, Hilden, Germany; AAGATCCTATCACAGGGCGAT), *AUF1* (Santa Cruz; sc‐37028), *DICER1* (Santa Cruz Biotechnologies, Dallas, Texas, USA; sc‐40489), *PRKAA1*, and *PRKAA2* (AMPKα1 and AMPKα2; both SMARTpools from Dharmacon) or a control siRNA (Qiagen; All Stars Negative Control). FLAG‐tagged plasmids containing p37, p40, p42, or p45 AUF1 isoforms were transfected together in equal concentrations (Fig. 2E) or individually for RIP experiments. Twenty‐four hours after transfection, HeLa cells were treated with 500 μm metformin or PBS in low serum media (0.1% FBS) for 24 h. HeLa cells were fractionated as described in Supporting Information.

WI‐38 and IMR‐90 cells were pretreated for 1 h with 500 μm metformin and then exposed to 10 Gy of ionizing radiation. Media and metformin was changed every 48 h, and RNA, protein, and SA‐β‐gal staining (described in Supporting Information) were performed 7 days after IR. To induce senescence in IDH4 cells, dexamethasone was removed and media changed to charcoal‐stripped FBS with or without 500 μm metformin. Media was changed every 48 h and after 7 days, RNA and protein were isolated, and SA‐β‐gal staining was performed.

### Reverse transcription (RT) followed by real‐time, quantitative (q)PCR analysis

RNA was reverse‐transcribed using the QuantiMir cDNA kit (System Biosciences, Mountain View, CA, USA) according to the manufacturer's instructions and real‐time, quantitative (q)PCR amplification of mRNA, rRNA, pri‐microRNA, and microRNA was carried out as explained in the Supporting Information.

### Immunoprecipitation, immunoblotting, and immunofluorescence

Mouse livers were homogenized in RIPA buffer containing protease and phosphatase inhibitors. Lysates were centrifuged, and equal protein concentrations of the supernatant were analyzed using SDS‐PAGE. For immunoprecipitation experiments, HeLa cells were serum‐starved in 0.1% FBS‐containing media for 18 h then treated for the indicated times with 500 μm metformin or PBS. Cells were lysed in a buffer containing TBS, 1% Triton‐X100, 2 mm EDTA, and phosphatase and protease inhibitors, clarified, and incubated with Gamma bind beads precoated with anti‐AUF1 antibodies (Cell Signaling, Danvers, MA, USA) for 1.5 h. After extensive washing, immunoprecipitations were analyzed by SDS‐PAGE. The antibodies used for immunoblotting are described in the Supporting Information.

AUF1 immunofluorescence in HeLa cells was analyzed as described in Supporting Information, using a Zeiss Observer D1 microscope with an AxioCam1Cc1 camera at a set exposure time.

### Immunoprecipitation of ribonucleoprotein (RNP) complexes

HepG2 cells were starved for 18 h and then treated in serum‐free media for 1 h with 500 μm metformin or PBS, and ribonucleoprotein (RNP) immunoprecipitation (RIP) assays were carried out as described previously (Yoon *et al*., 2014). To test AUF1 isoform binding to *DICER1* mRNA, HeLa cells were transfected with individual plasmids encoding FLAG‐tagged p37, p40, p42, or p45 AUF isoforms. RIP analysis is described in Supporting Information.

## Funding info

No funding information provided.

## Author contributions

NNH and MKE designed the study. NNH performed the experiments. AM‐M, MB, and RdC performed the metformin mouse study. MG provided reagents, PAR‐CLIP, and advice. ABZ and MKE are coprincipal investigators of HANDLS. DFD isolated RNA and performed primary miRNA RT–qPCR and target prediction analysis. YZ and KGB performed microarray analysis. NNH wrote the paper with input from all authors.

## Conflict of interest

The authors declare that they have no conflict of interest.

## Supporting information


**Fig. S1** AUF1 protein levels and subcellular localization in response to metformin.
**Fig. S2** RT‐qPCR analysis of miRNAs from liver of mice on standard diet, treated with metformin or on caloric restriction.
**Table S1** PAR‐CLIP analysis of AUF1 isoform binding to *DICER1* mRNA.
**Table S2** miRNA expression data from heat map.
**Table S3** Predicted, senescence‐related miRNA targets.
**Table S4** RT‐qPCR primers used in this study.
**Data S1** Supporting Experimental Procedures.
