# Supplementary material for: Metformin‐mediated increase in DICER1 regulates microRNA expression and cellular senescence
Source: Aging Cell. 2016 Mar 17;15(3):572–81. doi: 10.1111/acel.12469 (PMC4854919; doi:10.1111/acel.12469)
Supplement: Supplementary file 1 — Fig. S1 AUF1 protein levels and subcellular localization in response to metformin. Fig. S2 RT‐qPCR analysis of miRNAs from liver of mice on standard diet, treated with metformin or on caloric restriction. Table S1 PAR‐CLIP analysis of AUF1 isoform binding to DICER1 mRNA. Table S2 miRNA expression data from heat map. Table S3 Predicted, senescence‐related miRNA targets. Table S4 RT‐qPCR primers used in this study. Data S1 Supporting Experimental Procedures. [file ACEL-15-572-s001.pdf]

Supporting Information

Supporting Experimental Procedures

Human study participants and mouse models

The HANDLS study is approved by the Institutional Review Board of the National Institute of Environmental Health Sciences, National Institutes of Health. All of the participants signed a written informed consent document. To examine *DICER1* levels in non-diabetics, diabetics taking sulfonyureas (but not metformin) and diabetics taking metformin, individuals from HANDLS were chosen by creating a three-group match for age, sex, race and BMI (below or above obesity cut-off) (Table IB). BMI greater or equal to 30 kg/m^2 is classified as obese. Diabetes mellitus is defined by self-report, use of oral hypoglycemic agents or fasting blood sugar >125 mg/dl. Euglycemic patients have fasting glucoses of 99 and below.

DICER1 levels were quantified from PBMCs by RT-qPCR analysis blinded to group assignment and normalized to the average of *HPRT* mRNA and *UBC* mRNA expression levels. Previously, we found that these reference genes were the least variable in human PBMCs (Noren Hooten *et al.* 2010). We used separate mixed-model regressions to account for matching and to examine differences in expression among the three treatment groups by comparing non-diabetics to diabetics taking sulfonyureas (but not metformin) and non-diabetics to diabetics taking metformin. We used R to perform all analyses and we set statistical significance to $p<0.05$.

Frozen tissue from Martin-Montalvo et al. was used for the current study (Animal protocol #: 352-TGB-2015). Livers from C57BL/6 mice on metformin (0.1% w/w in diet), calorie restriction (60% daily food, AIN-93G, allotment compared to *ad lib* animals) or standard diet (AIN-93G diet) used for these studies were described previously (Martin-Montalvo *et al.* 2013). More information about animal procedures, housing and diets are detailed elsewhere (Martin-Montalvo *et al.* 2013).

Reverse transcription (RT) followed by real-time, quantitative (q)PCR analysis

Forward primers were designed to match the exact sequence of the mature miRNA and a universal reverse primer was provided by the manufacturer. For senescence experiments, RNA was reverse transcribed using random hexamers and reverse transcriptase. Specific primers were used to detect the levels of expressed mRNAs. All primer sequences are listed in Table S4. SYBR Green PCR master mix (Applied Biosystems) was used for real-time quantitative (q)PCR amplification using an Applied Biosytems7500 Real-Time PCR machine. For mouse studies, miRNAs were normalized to *U6* expression levels and mRNA levels were normalized to *Gapdh* mRNA levels. *GAPDH* mRNA levels were used to

normalize mRNA levels from human cell lines. *18S* was used for normalization in senescence experiments. Primer sequences are listed in Table S4.

For the analysis of mouse pri-miRNAs, RNA was reverse transcribed using the High-Capacity cDNA Reverse Transcription Kit (Life Technologies) according to the manufacturer's instructions. Mouse primary miRNAs (pri-miRNAs) were quantified using Taqman Pri-miRNA primers and probes for mmu-miR-92a-1 (Assay ID Mm03306814_pri) and mmu-miR-130a (ID Mm03306263_pri). Taqman Gene Expression Master Mix (Life Technologies) was used for real-time RT-PCR on an Applied Biosystems 7500 Real-Time PCR machine. Primary miRNAs were normalized to *Gapdh* expression levels.

Cell fractionation and antibodies used for immunoblotting

To fractionate HeLa or WI-38 cell nuclear and cytoplasmic compartments, cells serum-starved for 18 hrs were pre-treated with 5 μM Compound C or DMSO control for 1 hr and then treated for 1 hr with 500 μM metformin or PBS and then fractionated into cellular and nuclear compartments using the NE-PER kit from Thermo Scientific according to manufacturer's instructions. An additional wash of the nuclear pellet with cold PBS was included to prevent cytoplasmic contamination into the nuclear fraction. Samples were analyzed by SDS-PAGE and immunoblotted as described above.

For immunoblotting, we used antibodies that recognized AUF1 (Millipore; 07-260), p21 (Millipore; 05-345), p16 (BD Biosciences; 51-1325GR), DICER1 (sc-30226), GAPDH (sc-32233), HSP70 (Abcam; 2787), Phosph-AMPK-substrate motif (Cell Signaling; 5759), Phospho-Serine/Threonine (Abcam; 17464), lamin-B1 (sc-6216), and actin (sc-1616); 'sc' antibodies were from Santa Cruz Biotechnology. DICER1 protein levels were quantified from immunoblots using ImageJ and normalized to actin (n=8 mice per condition).

Immunoprecipitation of ribonucleoprotein (RNP) complexes

For ribonucleoprotein (RNP) immunoprecipitation (RIP) assays, cells were lysed for 10 min on ice in a buffer containing 20 mM Tris-HCl pH 7.5, 100 mM KCl, 5 mM $MgCl_2$, 0.5% NP-40, RNaseOUT and protease inhibitors and then centrifuged at 10,000 *g* for 15 min at 4°C. The supernatants were incubated with mouse IgG agarose beads (Sigma-Aldrich) that were precoated with anti-FLAG M2 antibodies or Gamma bind beads precoated with anti-AUF1 (Millipore; 07-260) or rabbit IgG (Santa Cruz Biotechnology) antibodies overnight at 4°C. After extensive washing with ice-cold NT2 buffer (50 mM Tris-HCl pH 7.5, 150 mM NaCl, 1 mM $MgCl_2$, 0.05% NP-40), the complexes were incubated with DNase I (RNase-free; Ambion) for 10 min at 30°C and subsequently with 0.1% SDS, Proteinase K (0.5 mg/ml)

for 15 min at 55°C to digest proteins present on the beads. RNA was extracted using acidic phenol, precipitated in the presence of glycoblue, and quantified by RT-qPCR analysis.

Immunofluorescence

HeLa cells plated on glass coverslips were serum-starved for 18 hrs and then treated for 1 hr with 500 μM metformin or PBS. Cells were fixed in 3.7% formaldehyde in PBS for 15 min, permeabilized with 0.5% Triton X-100 in TBS for 3 min, washed in TBS, and then blocked for 1 hr in TBS containing 1% BSA and 10% goat serum. Cells were incubated with anti-AUF1 antibodies (Millipore) for 1 hr, washed, and incubated with secondary antibodies (Thermo Fisher Scientific) for 1 hr, washed and then stained with DAPI. Fluorescent images were taken on a Zeiss Observer D1 microscope with an AxioCam1Cc1 camera at a set exposure time.

microRNA microarray

We analyzed global microRNA expression from livers of the same cohort of mice (n=5 per group) whose global gene expression profile was previously reported (Accesssion Number: GSE40936)(Martin-Montalvo *et al.* 2013). Total RNA including miRNAs was isolated using the Absolutely RNA miRNA Kit (Agilent) and analyzed using the Agilent Mouse miRNA Microarray 15.0. Microarray was performed and analyzed as previously described (Noren Hooten *et al.* 2013). Individual miRNAs with pairwise z-test p value $\leq$ 0.05, absolute value of Z ratio $\geq$1.5 , with fdr $\leq$ 0.3 were considered significantly changed. The microRNA microarray data can be accessed at GEO (Accession Number: GSE73393). miRNA expression information from heat map are in Table S2.

SA-β-Gal staining

Pre-senescent WI-38 cells at passage ~38 or IMR-90 cells at passage ~52, were transfected with control siRNA, *DICER1* siRNA, *PRKAA1* and *PRKAA2* (encoding AMPKα1 and AMPKα2) or pDESTmycDICER1 (Addgene). Eight hours later, HDFs were treated with either PBS or 500 μM metformin; 48 hrs later, the presence of senescence-associated β-galactosidase (SA-β-gal) in HDFs was assessed using a kit from Cell Signaling.

Supporting Figure Legends

Figure S1. AUF1 protein levels and subcellular localization in response to metformin. (A) AUF1 protein levels were assessed by immunoblotting liver lysates from mice treated with metformin (n=8 mice/group)

and from (B) serum-starved HeLa cells treated with metformin for 1 hr (n=3). (C) AUF1 protein levels were quantified from immunoblots and normalized to actin. Histogram represents the mean + SEM. (D) WI-38 cells were pretreated with Compound C for 1 hr or vehicle and were then subsequently treated with 500 μM metformin for 1 hr. Cells were fractionated into cytoplasmic and nuclear fractions and analyzed by immunoblotting with anti-AUF1, anti-Lamin B1 (nuclear marker) and anti-GAPDH antibodies (cytoplasmic marker).

Figure S2. RT-qPCR analysis of miRNAs from liver of mice on standard diet (SD), treated with metformin (Met) or on caloric restriction (CR). *$p<0.05$ by Student's t-test.

Table S1. PAR-CLIP analysis of AUF1 isoform binding to *DICER1* mRNA.

Table S2. miRNA expression data from heat map.

Table S3. Predicted, senescence-related miRNA targets.

Table S4. RT-qPCR primers used in this study.

Supporting References

Martin-Montalvo A, Mercken EM, Mitchell SJ, Palacios HH, Mote PL, Scheibye-Knudsen M, Gomes AP, Ward TM, Minor RK, Blouin MJ, Schwab M, Pollak M, Zhang Y, Yu Y, Becker KG, Bohr VA, Ingram DK, Sinclair DA, Wolf NS, Spindler SR, Bernier M , de Cabo R (2013). Metformin improves healthspan and lifespan in mice. *Nature communications*. **4**, 2192.

Noren Hooten N, Abdelmohsen K, Gorospe M, Ejiogu N, Zonderman AB , Evans MK (2010). microRNA expression patterns reveal differential expression of target genes with age. *PloS one*. **5**, e10724.

Noren Hooten N, Fitzpatrick M, Wood WH, 3rd, De S, Ejiogu N, Zhang Y, Mattison JA, Becker KG, Zonderman AB , Evans MK (2013). Age-related changes in microRNA levels in serum. *Aging (Albany NY)*. **5**, 725-740.

Figure S1.

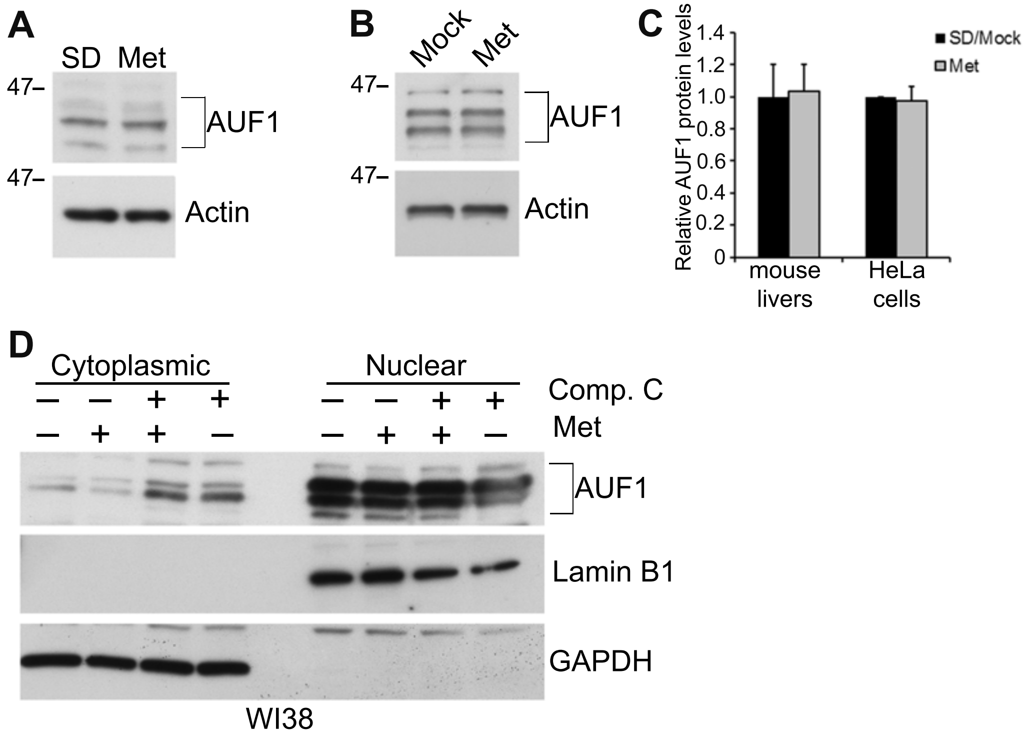
A
SD
Met
47–
AUF1
47–
Actin
B
Mock
Met
47–
AUF1
47–
Actin
C
Relative AUF1 protein levels
1.4
1.2
1.0
0.8
0.6
0.4
0.2
0
SD/Mock
Met
mouse livers
HeLa cells
D
Cytoplasmic
Nuclear
Comp. C
Met
AUF1
Lamin B1
GAPDH
WI38

Figure S2

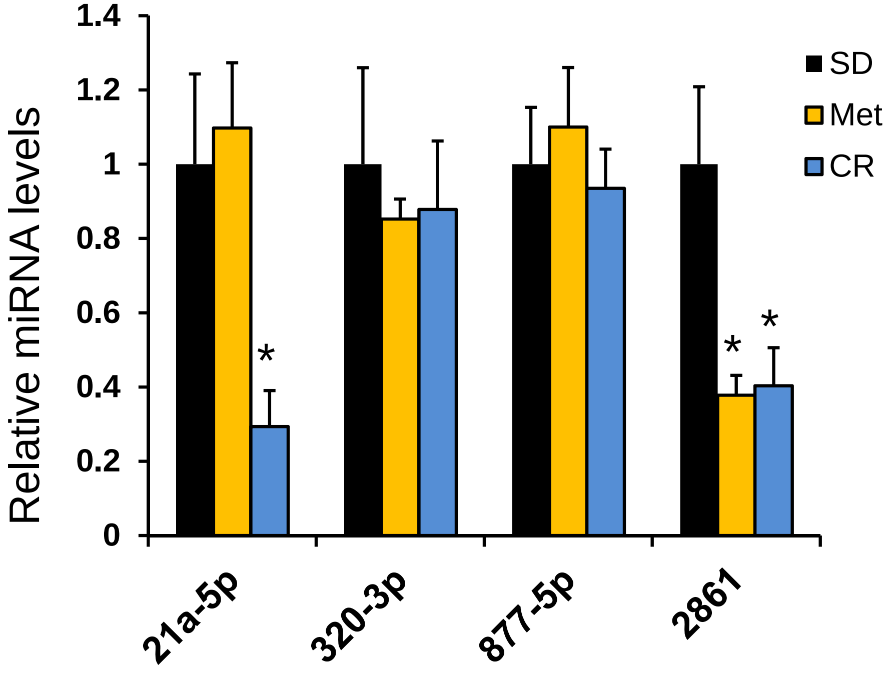
Relative miRNA levels
1.4
1.2
1
0.8
0.6
0.4
0.2
0
SD
Met
CR
*
*
*
21a-5p
320-3p
877-5p
2861

Table S1. PAR-CLIP analysis of AUF1 isoform binding to DICER1 mRNA. Datasets are from Yoon, JH et al., 2014; Nature Communications, 5, 5248.

Isoforms	Chromoso	Strand	Start	End	GroupID	GroupSequence	Read Count	Conversion LocationCount	Conversion EventCount	Fraction of T to C conversion	Transcript Location	TranscriptID	Gene Name
p37	-	-	-	-	-	-	-	-	-	-	-	-	-
p40	chr14	-	95553660	95553690	G3602	ATATTTCTTTTCTTTTTTCTTTTATATTACG	5	0	0	0	3'utr	ENST00000343455	DICER1
p40	chr14	-	95554015	95554038	G3603	TTTTAAAATTTCTTTTATAATATG	5	1	5	1	3'utr	ENST00000343455	DICER1
p40	chr14	-	95555346	95555380	G3604	TAACTATAATACCTCTTAATTTACCTTTTTAAAAG	10	0	0	0	3'utr	ENST00000343455	DICER1
p40	chr14	-	95570387	95570421	G3605	CAAATCTTTCATCTCAATTTCTAACTCCTCTTCAG	6	0	0	0	coding	ENST00000541352	DICER1
p42	chr14	-	95553660	95553685	G11349	TCTTTTCTTTTTTCTTTTATATTACG	7	1	7	1	3'utr	ENST00000343455	DICER1
p45	chr14	-	95587720	95587739	G10898	CCCATTACTATTAACTCCAG	5	2	5	1	intron	ENST00000343455	DICER1
p45	chr14	-	95552935	95552969	G10896	TAAATATATTTTAATCATTACTTCTTTTTTTCTAG	5	2	4	0.8	3'utr	ENST00000343455	DICER1
p45	chr14	-	95589019	95589046	G10899	ACTATCTAATCCAACCCACTTATTTTTG	6	1	4	0.666666667	intron	ENST00000343455	DICER1
p45	chr14	-	95555697	95555749	G10897	CACCTTTACCCTTAGTCTCCTCACATAAATTTCTTACTATACTTTTCATAATG	6	3	3	0.5	3'utr	ENST00000343455	DICER1

Table S2. miRNA expression data from heat map.

Symbol	(zratio) CR-SD	(zratio) CR-MET	(zratio) MET-SD	(fold change) CR-SD	(fold change) CR-MET	(fold change) MET-SD	(p-value) CR-SD	(p-value) CR-MET	(p-value) MET-SD
mmu-miR-720_v18.0	-7.84	-7.69	-3.74	-3.29	-2.6	-1.27	0	0.0097	0.398
mmu-miR-1937c_v16.0	-6.5	-5.57	-3.85	-2.6	-2.1	-1.24	0	0.0062	0.2397
mmu-miR-1937b_v16.0	-5.53	-5.8	-3.32	-3.35	-2.51	-1.34	0	0.0034	0.2914
mmu-miR-5097	-5.25	-7.16	-3.64	-3.1	-2.39	-1.3	0	0.0069	0.198
mmu-miR-21a-5p	-6.69	-8.33	1.27	-2.43	-2.22	-1.09	0.003	0.0064	0.4056
mmu-miR-192-5p	-3.97	-6.18	2.16	-1.65	-1.84	1.11	0.0531	0.0173	0.1303
mmu-miR-148a-3p	-3.95	-5.12	0.86	-1.7	-1.76	1.03	0.0058	0.0048	0.809
mmu-miR-15a-5p	-2.82	-4.38	1.6	-1.52	-1.66	1.09	0.0109	0.0027	0.3547
mmu-miR-1939_v16.0	-2.55	-4.98	1.49	-1.91	-2.13	1.11	0	0.0005	0.8384
mmu-let-7f-5p	-2.49	-4.58	2.3	-1.49	-1.63	1.09	0.0981	0.0168	0.1679
mmu-miR-194-5p	-2.22	-4.18	2.21	-1.42	-1.61	1.14	0.0731	0.0177	0.0584
mmu-miR-26b-5p	-2.7	-3.94	1.43	-1.49	-1.6	1.07	0.0358	0.0126	0.3518
mmu-miR-29c-3p	-2.47	-3.7	1.55	-1.46	-1.57	1.08	0.0213	0.0097	0.3293
mmu-miR-101b-3p	-2.51	-4.38	-0.3	-1.65	-1.63	-1.02	0.0019	0.0043	0.3179
mmu-miR-29b-3p	-2.16	-4.89	-0.3	-1.75	-1.72	-1.02	0.0057	0.0084	0.597
mmu-miR-19b-3p	-1.84	-3.54	0.32	-1.27	-1.31	1.03	0.0018	0.0032	0.6805
mmu-miR-30e-5p	-1.61	-3.35	0.6	-1.26	-1.32	1.05	0.0001	0.0001	0.9368
mmu-miR-193a-3p	-1.79	-3.1	-0.02	-1.26	-1.23	-1.02	0.0055	0.0337	0.1081
mmu-miR-126-3p	-2.14	-2.79	0.9	-1.33	-1.42	1.06	0.0576	0.0267	0.3218
mmu-miR-101a-3p	-1.81	-2.88	0.79	-1.28	-1.32	1.03	0.002	0.0034	0.619
mmu-miR-30c-5p	-1.67	-3.01	1.04	-1.26	-1.34	1.07	0.0637	0.0276	0.4185
mmu-miR-30b-5p	-1.64	-3.14	1.21	-1.39	-1.49	1.07	0.0562	0.0242	0.327
mmu-let-7g-5p	-1.94	-3.02	1.92	-1.37	-1.43	1.05	0.0559	0.0145	0.601
mmu-miR-16-5p	-1.91	-3.51	1.66	-1.34	-1.49	1.11	0.0767	0.0156	0.2462
mmu-miR-30a-5p	-1.74	-2.2	0.54	-1.37	-1.36	-1.01	0.0333	0.0442	0.6356
mmu-miR-466f-3p	-1.57	-1.78	0.24	-1.23	-1.28	1.04	0.0412	0.0007	0.8908
mmu-miR-106b-5p	-1.46	-1.92	0.38	-1.18	-1.24	1.06	0.0013	0.0006	0.6575
mmu-miR-107-3p	-1.32	-1.65	0.57	-1.26	-1.31	1.04	0.0475	0.003	0.676
mmu-miR-152-3p	-1.19	-1.62	0.82	-1.14	-1.23	1.08	0.0013	0.0001	0.1935
mmu-miR-199a-3p	-1.07	-1.65	0.9	-1.13	-1.23	1.09	0.0592	0.0169	0.3654
mmu-miR-142-3p	-1.07	-1.73	0.87	-1.15	-1.25	1.09	0.0039	0.0007	0.3468
mmu-miR-20a-5p	-1.21	-2.24	0.74	-1.18	-1.27	1.08	0.0629	0.0234	0.4743
mmu-miR-27b-3p	-1.07	-1.87	1.28	-1.23	-1.29	1.05	0.0289	0.0136	0.7323
mmu-miR-10a-5p	-0.97	-1.85	1.13	-1.1	-1.23	1.11	0.0984	0.0123	0.0443
mmu-miR-125a-3p	-0.94	-1.54	1.23	-1.1	-1.26	1.14	0	0.0088	0.372
mmu-miR-195a-5p	-0.89	-1.52	1.32	-1.09	-1.22	1.13	0.1009	0.0114	0.0259
mmu-miR-212-3p	-1.59	-3.09	-1.33	-1.7	-1.46	-1.17	0	0.0111	0.185
mmu-miR-203-3p	-1.55	-1.13	-1.24	-1.2	-1.17	-1.02	0.0008	0.0126	0.0674
mmu-miR-34a-5p	-0.9	-2.37	2.5	-1.15	-1.31	1.14	0.0027	0	0.2201
mmu-miR-26a-5p	-0.24	-1.48	2.87	-1.11	-1.32	1.19	0.6848	0.0129	0.0014
mmu-miR-130a-3p	0.2	-1.99	3.79	-1.14	-1.36	1.19	0.3217	0.0036	0.0007
mmu-miR-139-5p	0.9	-0.16	1.83	1.16	1.01	1.15	0	0.2266	0
mmu-miR-92a-3p	2.15	0.2	2.74	1.24	1.06	1.18	0	0	0
mmu-miR-338-5p	1.68	1.15	1.53	1.29	1.13	1.14	0	0.0022	0.0388
mmu-miR-1187	1.92	1.49	1.15	1.3	1.21	1.08	0.0054	0.0101	0.5459
mmu-let-7c-5p	1.92	0.03	3.94	1.07	-1.12	1.2	0.0076	0.5676	0.0192
mmu-miR-125b-5p	2.08	0.06	3.91	1.18	-1.01	1.2	0.0003	0.1207	0.0037
mmu-let-7b-5p	2.4	0.36	4.02	1.1	-1.06	1.16	0.0035	0.1185	0.062
mmu-miR-122-5p	2.26	-0.2	4.67	-1.01	-1.22	1.21	0	0.1491	0.0005
mmu-miR-1944_v16.0	1.24	1.5	-0.41	1.06	1.1	-1.03	0.0415	0.0053	0.2412
mmu-miR-1906	1.25	1.5	-0.5	1.16	1.17	-1.01	0	0	0.0001

mmu-miR-574-5p	2.12	2.06	0.52	1.32	1.23	1.07	0.0097	0.0085	0.7404
mmu-miR-2134_v15.0	2.27	2.31	-0.72	1.08	1.16	-1.08	0.0233	0.0024	0.3523
mmu-miR-2133_v15.0	2.57	1.81	-0.28	1.11	1.21	-1.09	0.0116	0.0024	0.3287
mmu-miR-2145_v16.0	1.73	3.03	-2.85	-1.07	1.36	-1.45	0.6466	0	0.0361
mmu-miR-2141_v15.0	2.16	2.38	-1.45	1.01	1.17	-1.16	0.0415	0.0017	0.1367
mmu-miR-2135_v15.0	3.36	3.08	-0.45	1.2	1.28	-1.07	0.0214	0.0098	0.4382
mmu-miR-2146_v15.0	4.31	2.92	1.58	1.08	1.11	-1.03	0.0064	0.0072	0.7275
mmu-miR-2132_v15.0	-1.51	0.73	-8.38	-1.64	1.08	-1.78	0.1645	0.0077	0.0057
mmu-miR-2861	-0.79	0.61	-4.41	-1.16	1.12	-1.3	0.1662	0.0017	0.0001
mmu-miR-671-5p	-0.12	0.52	-1.52	1.01	1.05	-1.03	0.5533	0.0004	0.0015
mmu-miR-3072-5p	0.05	0.7	-1.53	1.02	1.06	-1.04	0.8562	0.0027	0.0001
mmu-miR-1897-3p	0.06	0.96	-2.1	1.02	1.09	-1.07	0.7797	0	0
mmu-miR-2137	0.82	0.6	-1.56	1.05	1.16	-1.1	0.1774	0	0.0037

Table S3: Predicted, senescence-related miRNA targets

microRNA	Predicted Targets	Previously Validated
has-miR-20a-5p	*APP, ATG5, ATG7, ATMIN, BTG3, CCND1, CDKN1A, COPS2, DMTF1, E2F1, E2F3, EIF4E, EP300, EREG, ESR1, FLT1, KCNA1, MAP3K7, MAPK1, NPM1, PBRM1, PDPK1, PTEN, RBL1, RBL2, RUNX1, SMAD7, SMURF2, SRSF2, SUV420H1, TBX3, TCEB3, TFGBR2, TP73, VHL, ZEB2*	*CDKN1A* (Inomata *et al.* 2009), *E2F1* (Sylvestre *et al.* 2007), *PTEN* (Trompeter *et al.* 2011), *RBL2* (Wang *et al.* 2008)
hsa-miR-34a-5p	*ATG5, ATMIN, BCL6, CBX2, CDKN1C, CSNK2A2, DMPK, E2F3, ERBB2, HLX, HSPA1A, HSPA1B, KCNA1, MAP2K1, MET, PRKD1, SIRT1, TBX2, TP73*	*SIRT1* (Badi *et al.* 2014; Ye *et al.* 2015)
hsa-miR-130a-3p	*CCNA2, CDKN1A, CPEB1, CSNK2A1, DICER1, DPY30, EREG, ESR1, GADD45A, KDM2A, MAPK1, MET, MITF, PTEN, SMURF2, SRSF2, TGFBR2, UBC, ZEB2*	*DICER1* (Kovaleva *et al.* 2012; He *et al.* 2014)
hsa-let-7c-5p	*ARID3A, BCL2L1, CASP3, CCND1, CDKN1A, COL4A2, CPEB1, CSNK2A1, DICER1, DUSP1, E2F6, ELF4, HELLS, MAGEA2, MAGEA2B, MTF2, NRAS, RB1, TP53, ULK3*	*BCL2L1* (Shimizu *et al.* 2010),
hsa-miR-125b-5p	*ARID3A, BRCA1, CBX7, CDC14B, CSNK2A1, CSNK2A2, DDB2, DICER1, DPY30, KCNA1, MAP2K7, MAPK12, PHC2, RAF1, TBX2, TP73, UBN1*	*ARID3A* (Puissegur *et al.* 2012)
hsa-miR-133a-5p	*AHCY, AKT1, ARF1, ATG10, ATG5, ATG7, ATMIN, BMI1, BMP7, BRD7, CASP3, CBX2, CDC14B, CDKN2B, CHRDL1, CSNK2A1, DEK, DMTF1, E2F3, EGFR, EIF4E, EIF4EBP1, ESR1, HGF, HIF1A, IFNB1, ING1, KAT6A, KDM5A, KRAS, KSR1, LIN9, MAP2K3, MAP2K6, MAP3K7, MAPK1, MAPKAPK5, MDM2, ME2, MELK, MTF2, NSMCE2, NUDT1, OPA1, PDPK1, PPARD, PSMB5, RARA, RELA, RUNX1, SIRT1, SIRT2, SOX9, SUV420H1, TCF3, TERT, TFTBR2, THRB, TM4SF1, TP53, TP53BP2, TPP2, UIMC1, VCAN, WNT1*	N/A
hsa-miR-92a-3p	*ARF1, CDKN1C, COPS2, DUSP1, EGFR, HGF, IGFBP7, KCNA1, KDM2A, MAP2K4, MDM2, MITF, MORC3, MTF2, PBRM1, PTEN, SIRT6, SMAD7, SUV420H1, TOP1, TP63*	*PTEN* (Ke *et al.* 2015)
hsa-miR-2861	*BRF1, KDM2A, MAP2K7, PAX8, PBRM1, PTPRC, SIRT1, TP73*	N/A

References

Badi I, Burba I, Ruggeri C, Zeni F, Bertolotti M, Scopece A, Pompilio G, Raucci A (2014). MicroRNA-34a Induces Vascular Smooth Muscle Cells Senescence by SIRT1 Downregulation and Promotes the Expression of Age-Associated Pro-inflammatory Secretory Factors. *The journals of gerontology. Series A, Biological sciences and medical sciences.*

He L, Wang HY, Zhang L, Huang L, Li JD, Xiong Y, Zhang MY, Jia WH, Yun JP, Luo RZ, Zheng M (2014). Prognostic significance of low DICER expression regulated by miR-130a in cervical cancer. *Cell Death Dis*. **5**, e1205.

Inomata M, Tagawa H, Guo YM, Kameoka Y, Takahashi N, Sawada K (2009). MicroRNA-17-92 down-regulates expression of distinct targets in different B-cell lymphoma subtypes. *Blood*. **113**, 396-402.

Ke TW, Wei PL, Yeh KT, Chen WT, Cheng YW (2015). MiR-92a Promotes Cell Metastasis of Colorectal Cancer Through PTEN-Mediated PI3K/AKT Pathway. *Ann Surg Oncol*. **22**, 2649-2655.

Kovaleva V, Mora R, Park YJ, Plass C, Chiramel AI, Bartenschlager R, Dohner H, Stilgenbauer S, Pscherer A, Lichter P, Seiffert M (2012). miRNA-130a targets ATG2B and DICER1 to inhibit autophagy and trigger killing of chronic lymphocytic leukemia cells. *Cancer Res*. **72**, 1763-1772.

Puissegur MP, Eichner R, Quelen C, Coyaud E, Mari B, Lebrigand K, Broccardo C, Nguyen-Khac F, Bousquet M, Brousset P (2012). B-cell regulator of immunoglobulin heavy-chain transcription (Bright)/ARID3a is a direct target of the oncomir microRNA-125b in progenitor B-cells. *Leukemia*. **26**, 2224-2232.

Shimizu S, Takehara T, Hikita H, Kodama T, Miyagi T, Hosui A, Tatsumi T, Ishida H, Noda T, Nagano H, Doki Y, Mori M, Hayashi N (2010). The let-7 family of microRNAs inhibits Bcl-xL expression and potentiates sorafenib-induced apoptosis in human hepatocellular carcinoma. *J Hepatol*. **52**, 698-704.

Sylvestre Y, De Guire V, Querido E, Mukhopadhyay UK, Bourdeau V, Major F, Ferbeyre G, Chartrand P (2007). An E2F/miR-20a autoregulatory feedback loop. *J Biol Chem*. **282**, 2135-2143.

Trompeter HI, Abbad H, Iwaniuk KM, Hafner M, Renwick N, Tuschl T, Schira J, Muller HW, Wernet P (2011). MicroRNAs MiR-17, MiR-20a, and MiR-106b act in concert to modulate E2F activity on cell cycle arrest during neuronal lineage differentiation of USSC. *PLoS One*. **6**, e16138.

Wang Q, Li YC, Wang J, Kong J, Qi Y, Quigg RJ, Li X (2008). miR-17-92 cluster accelerates adipocyte differentiation by negatively regulating tumor-suppressor Rb2/p130. *Proc Natl Acad Sci U S A*. **105**, 2889-2894.

Ye Z, Fang J, Dai S, Wang Y, Fu Z, Feng W, Wei Q, Huang P (2015). MicroRNA-34a induces a senescence-like change via the down-regulation of SIRT1 and up-regulation of p53 protein in human esophageal squamous cancer cells with a wild-type p53 gene background. *Cancer Lett*.

Table S4. Real-time RT-PCR primers used in this study.

Human Primers	Forward	Reverse
18S	CCCTATCAACTTTCGATGGTAGTCG	CCAATGGATCCTCGTTAAAGGATTT
CXCL1	GAAAGCTTGCCTCAATCCTG	CACCAGTGAGCTTCCTCCTC
CXCL2	AACTGCGCTGCCAGTGCT	CCCATTCTTGAGTGTGGCTA
DICER1	TTAACCTTTTGGTGTTTGATGAGTGT	GCGAGGACATGATGGACAATT
DROSHA	GGCCCGAGAGCCTTTTATAG	TGCACACGTCTAACTCTTCCAC
GAPDH	GCTCCTCCTGTTCGACAGTCA	ACCTTCCCCATGGTGTCTGA
HNRNPD (AUF1)	GATCCTAAAAGGGCCAAAGC	GTTGTCCATGGGGAGCTCTA
HPRT1	AGATGGTCAAGGTCGCAAGCT	GGGCATATCCTACAACAAACTTGTC
IL6	CCGGGAACGAAAGAGAAGCT	GCGCTTGTGGAGAAGGAGTT
IL8	CTTTCCACCCCAAATTTATCAAAG	CAGACAGAGCTCTCTTCCATCAGA
UBC	ATTTGGGTCGCGGTTCTTG	TGCCTTGACATTCTCGATGGT

Mouse Primers	Forward	Reverse
Dicer1	CAACACTGCCATTGGACACA	TCAGGCAATTCTCGGGTTCT
Gapdh	TGTGTCCGTCGTGGATCTGA	CCTGCTTCACCACCTTCTTGAT

**miRNA forward primers (Human miRNAs examined in Table 1 were homologous to mouse miRNAs)**	
hsa-miR-147a	GTGTGTGGAAATGCTTCTGC
hsa-miR-574-5p	TGAGTGTGTGTGTGTGAGTGTGT
mmu-let-7c-5p	TGAGGTAGTAGGTTGTATGGTT
mmu-miR-106b-5p	TAAAGTGCTGACAGTGCAGAT
mmu-miR-125b-5p	TCCCTGAGACCCTAACTTGTGA
mmu-miR-130a-3p	CAGTGCAATGTTAAAAGGGCAT
mmu-miR-133a-5p	GCTGGTAAAATGGAACCAAAT
mmu-miR-1897-3p	TCAACTCGTTCTGTCCGGTGAG
mmu-miR-20a-5p	TAAAGTGCTTATAGTGCAGGTAG
mmu-miR-21a-5p	TAGCTTATCAGACTGATGTTGA
mmu-miR-2861	GGGGCCTGGCGGCGGGCGG
mmu-miR-320-3p	AAAAGCTGGGTTGAGAGGGCGA
mmu-miR-34a-5p	TGGCAGTGTCTTAGCTGGTTGT
mmu-miR-877-5p	GTAGAGGAGATGGCGCAGGG
mmu-miR-92a-3p	TATTGCACTTGTCCCGGCCTG
U6	CACCACGTTT ATACGCCGGTG
